# Ocular involvement in melioidosis: a 23-year retrospective review

**DOI:** 10.1186/s12348-018-0147-6

**Published:** 2018-03-27

**Authors:** Sasi Yaisawang, Somkiat Asawaphureekorn, Ploenchan Chetchotisakd, Surasakdi Wongratanacheewin, Peerapat Pakdee

**Affiliations:** 1 Department of Ophthalmology, Faculty of Medicine, Khon Kaen University, Khon Kaen, Thailand; 2 Department of Medicine, Faculty of Medicine, Khon Kaen University, Khon Kaen, Thailand; 3 Melioidosis Research Center, Khon Kaen University, Khon Kaen, Thailand; 4 Department of Microbiology, Faculty of Medicine, Khon Kaen University, Khon Kaen, Thailand; 5 Department of Ophthalmology, Khon Kaen Hospital, Khon Kaen, Thailand

**Keywords:** Melioidosis, *Burkholderia pseudomallei*, Glanders, Orbital cellulitis, Endophthalmitis

## Abstract

**Background:**

Ocular involvement in melioidosis is rare and has devastating outcomes. Although there have been few reports on the condition, Khon Kaen, a city in northeast Thailand, has been called the “capital of melioidosis” due to the high prevalence of the condition in the region. We retrospectively reviewed all admitted cases of melioidosis with ocular involvement from the two largest hospitals in Khon Kaen. We reviewed cases from Srinagarind Hospital (a university hospital) of patients admitted between 1993 and 2016 and from Khon Kaen Hospital (a provincial hospital) of patients who presented from 2012 to 2016.

**Results:**

We identified 16 cases of ocular involvement. Eight of these cases were proven from positive culture, and the remaining eight were implied from high melioidosis titer. The prevalence was estimated as being from 0.49 to 1.02%. Most patients had underlying diseases (14, 88%), of which diabetes mellitus was the most prevalent (12, 75%). Nine cases (56%) were part of disseminated septicemia. Patients suffered from blindness in 11 (73%) of the 15 cases in which visual acuity was recorded. Orbital cellulitis was the most common manifestation (7, 44%) followed by endophthalmitis (4, 25%). Interestingly, all patients with necrotizing fasciitis (100%) developed septic shock as a consequence. In most of the cases, patients underwent surgery (13, 81%) including incision and drainage, debridement, and pars plana vitrectomy. Despite appropriate management, the visual outcomes were disappointing (9, 64%).

**Conclusion:**

To summarize, ocular melioidosis is a highly destructive disease. Early detection and prompt surgical management may reduce morbidity and mortality from septic shock.

## Background

Melioidosis is caused by a gram-negative, motile, non-spore forming facultative anaerobic bacillus known as *Burkholderia pseudomallei.* The organism is found in soil and surface water and is widely distributed in Southeast Asia, especially in northeast Thailand and northern Australia [1].

Melioidosis presents with broad spectrums of clinical presentations and organ involvement. However, there are few case reports of ocular involvement in melioidosis, and most of these are single-case report or small case series.

In northeast Thailand, there are around 2000 culture-positive melioidosis cases per year [2]. Khon Kaen, one of the largest cities in northeast Thailand, has been called “the capital of melioidosis” due to the high prevalence of the disease in the region. Ocular involvement in these cases has not been investigated. The primary objective of this study was to estimate the prevalence and investigate ocular manifestations of melioidosis in Khon Kaen. Management and visual outcomes in these patients were also reviewed.

## Results

We identified 16 cases of ocular involvement, 13 out of the 1270 melioidosis cases admitted to Srinagarind Hospital (prevalence 1.02%; 95% confidence interval from 0.58 to 1.76%) and three out of the 607 admitted cases at Khon Kaen Hospital (prevalence 0.49%; 95% confidence interval from 0.10 to 1.51%). Overall, the estimated prevalence of ocular involvement in cases of melioidosis was from 0.49 to 1.02% (Table 1).

**Table 1:** Data collection and prevalence (95%CI) calculation

Tertiary hospital	Srinagarind University Hospital	Khon Kaen Provincial Hospital	Total from all data	Total from 2012 to 2016
Date	January 1993 to April 2016	January 2012 to April 2016	January 1993 to April 2016	January 2012 to April 2016
Length	23 years and 4 months	4 years and 4 months	23 years and 4 months	4 years and 4 months
Total melioidosis (cases)	1270	607	1877	859
Total ocular involvement (cases)	13	3	16	8
Prevalence (95% CI)	1.02% (0.58, 1.76%)	0.49% (0.10, 0.51%)	0.85% (0.51, 1.39%)	0.93% (0.44, 1.86%)

Of those 16 cases, there were 8 with positive cultures. In the remaining eight cases, melioidosis was implied from the high titer for melioidosis in the bloodstream. Clinical descriptions of all cases are summarized in Table 2.

**Table 2:** Clinical descriptions of all cases

Year	Age	Sex	Occupation	Symptom	Laterality	Initial VA	Ocular positive finding	Ocular diagnosis	Risk factor	Type of melioidosis	Primary organ	Associated symptoms	Investigations	Treatments	Outcomes
2007	70	F	None	Progressive painful proptosis with fever 10 days, S/P IV cloxacillin at provincial hospital (onset = 10 days)	OD	No LP	Complete ptosis, mark eyelid swelling, IOP 21/13, marked chemosis, clear cornea, no C/F, positive RAPD, EOM 0% all direction, pale disc with choroidal fold	Orbital cellulitis	History of eye scratching with dirty hand	Disseminated	Eye	Acute sphenoidal sinusitis, meningitis, septic arthritis, melioidosis septicemia	Hemoculture: *B. pseudomellei*, LP: eosinophilic meningitis, MRI orbit: right orbital cellulitis with extraconal abscess latero-superior aspect	FESS, I&D, IV ceftazidime then oral bactrim, tarsorhaphy	VA no LP, limit EOM 90% at lateral gaze OD, other EOM are full, normal anterior segment, pale disc, attenuated vessel (No LP at initial)
2007	64	M	Farmer	Progressive proptosis 10 days PTA, S/P IV ceftazidime, IV clindamycin at provincial hospital	OS	CF 2 ft	Lid swelling, proptosis, chemosis, clear cornea, no C/F, positive RAPD, EOM 10% all direction	Orbital cellulitis	DM without DR, CKD	Disseminated	Eye	Pansinusitis, subcutaneous abscess at inferolateral of the eye	Hemoculture: *B. pseudomellei*, pus culture: *B. pseudomellei*. CT orbit: pansinusitis with severe orbital cellulitis	I&D, FESS, IV ceftazidime then oral bactrim, topical antibiotic	VA 6/24, VA with pinhole 6/12, less chemosis, less proptosis, normal anterior segment, EOM limit at downgaze
				(onset = 10 days)											(improve)
2008	61	M	Farmer	Progressive painful visual loss 2 weeks	OS	No LP	Generalized bedewing cornea, hypopyon 2-mm, shallow AC, C/F 3+/2+, positive RRAPD, EOM 50% all direction, B scan: generalized vitreous opacity, intra-op findings: dense vitreous abscess, subretinal abscess rupture to vitreous	Panophthalmitis	DM without DR, CKD	Localized	Eye		Hemoculture: no growth, melioid titer 1:5122, CT orbit: swelling of periorbital tissue	PPV, ECCE, topical vancomycin, topical ceftazidime, oral bactrim	VA no LP, conjunctival less chemosis, AC deep with plasmoid and hyphema, no record about posterior segment
				(onset = 2 weeks)											(No LP at initial)
2008	51	F	Teacher	Fever with dyspnea 12 days PTA, left eye inflammation was found during admission	OS	HM, good PJ	Corneal bedewing, C/F 4+/4+, positive RRAPD, intra-op findings: attenuated vessels, subretinal gliosis, shallow RD	Endogenous endopthalmitis	DM without DR	Disseminated	Hematogenous	Pulmonary edema	Hemoculture: no growth, melioid titer 1:5122, MRI orbit: preseptal cellulitis	PPV with silicone oil, ECCE, IV ceftazidime then oral bactrim, topical vancomycin, topical ceftazidime	VA HM poor PJ, AC deep with plasmoid, attach retina
				(onset = NA)											(stable)
2009	39	F	Farmer	Eye pain with fever 2 weeks	OD	6/24	Marked eyelid swelling and erythema, fluctuation, no discharge, clear cornea, no C/F, negative RAPD, normal posterior segment	Preseptal cellulitis	DM without DR	Multifocal	Eye	Pneumonia, subcutaneous abscess at right thigh	Pus culture: *B. pseudomellei*, melioid titer 1/640, hemoculture: NG, CT orbit: preseptal cellulitis	I&D upper eyelid, I&D right thigh, oral bactrim	VA 6/9, normal anterior and posterior segment
				(onset = 2 weeks)											(improve)
2011	46	M	Labor	Fever with constitutional symptoms 2 weeks then visual loss 3 days	OS	CF 2 ft	Conjunctival chemosis, corneal stromal edema, hypopyon, hyphema, C/F 4+/2+, retinal infiltration	Endogenous endophthalmitis	DM without DR	Multifocal	Hematogenous	Liver abscesses, splenic abscess	Hemoculture: no growth, melioid titer 1:5122, CT abdomen: multiple liver abscesses, splenic abscess	IV ceftazidime then oral bactrim	VA 3/60, VA with pinhole 4/60, contracted hypopyon, vitreous opacity grade 1
				(onset = 3 days)											(improve)
2011	43	F	Farmer	Painful proptosis 8 days PTA, S/P IV antibiotic at provincial hospital then alteration of consciousness 1 day	OS	Not done due to alteration of consciousness	Necrotizing fasciitis at left upper eyelid size 1 × 8 cm, purulent discharge, ciliary injection, clear cornea, no C/F, clear vitreous	Orbital cellulitis, necrotizing fasciitis	First dx DM without DR	Disseminated	Eye	Pansinusitis, melioidosis septic shock	Hemoculture: *B. pseudomellei*, pus culture: *B. pseudomellei*, CT orbit: orbital abscess at the superomedial wall of orbit, medial rectus muscle, lateral rectus muscle	Debridement of necrotic wound, IV ceftazidime then oral bactrim	Good wound, less swelling, no record about VA
				(onset = 8 days)											(NA)
2011	46	M	Labor	Painless visual loss 1 month PTA then painful proptosis 2 days	OS	LP, poor PJ	IOP 32, bedewing cornea, hypopyon with plasmoid in AC, negative RAPD, intra-op finding: subretinal abscess	Endogenous endopthalmitis	DM without DR	Multifocal	Eye, liver	Liver abscess	Hemoculture: NG, melioid titer 1:640	PPV with silicone oil, oral bactrim	Painful red eye 1 week after discharge, VA no LP, IOP 40, shallow AC, iris bombe end up with enucleation, intra-op finding: flank pus in the vitreous cavity
				(onset = 1 month)											(enucleated)
2012	63	M	Farmer	Painful proptosis 2 weeks	OD	20/200	Marked eyelid swelling, no discharge, conjunctival injection, keratic precipitates at the cornea, peripheral synechiae 360 degrees, C/F 4+/2+, vitreous opacity grade 4	Panuveitis, preseptal cellulitis	MDS, leukemia	Disseminated	Eye	Spondylodiscitis, epidural and paravertebral abscess	Hemoculture: no growth, melioid titer 1: 5120	IV ceftazidime, 1% prednisolone acetate eye drop RE qid	VA 20/200, peripheral synechiae 360 degrees, vitreous opacity grade 1
				(onset = 2 weeks)											(stable)
2012	54	M	Farmer	Fever with left side headache 1 week PTA then left facial edema 5 days PTA then painful proptosis 3 days	OS	No LP	Marked eyelid swelling, erythema and tender, copious pus and discharge, marked chemosis, clear cornea, no C/F, positive RRAPD, B scan: vitreous opacity, intra-op finding: pus 1 ml in the vitreous cavity, flame shape hemorrhage, disc swelling, venous congestion, drusen	Orbital cellulitis	DM without DR	Disseminated	Maxillary sinus	Maxillary sinusitis, melioidosis septicemia	Hemoculture: *B. pseudomellei*, pus culture: *B. pseudomellei*, CT orbit: maxillary sinusitis	I&D, orbital decompression, IV ceftazidime then oral bactrim	VA no LP, less swelling periorbital area, conjunctival chemosis, normal anterior segment, limit EOM all direction, fundus: disc swelling, flame shape hemorrhage
				(onset = 1 week)											(No LP at initial)
2012	65	M	House keeper	Fever with chill 3 days PTA then alteration of consciousness 1 day, then right upper eyelid swelling at the emergency department	OD	6/6	Upper eyelid swelling, erythema and tender, conjunctival chemosis, clear cornea, no C/F, negative RAPD, normal posterior segment, full EOM	Preseptal cellulitis	DM without DR	Disseminated	Hematogenous	–	Hemoculture: no growth, melioid titer 1:640	IV ceftazidime then oral azithromycin	VA 6/6, no lid swelling, mild erythema, normal anterior and posterior segment
				(onset = < 1 day)											(improve)
2013	57	M	Thai massager	Pain at the left temporal area 3 week PTA then painful proptosis 3 days, S/P IV antibiotic at primary care hospital, S/P I&D temporal space abscess at provincial hospital	OS	20/200	Proptosis, chemosis, clear cornea, no C/F, negative RAPD, EOM 10-20% all direction, fundus: macular striae, mild pale disc	Orbital cellulitis	DM without DR	Localized	Temporal space abscess	Temporal space abscess, subperiosteal abscess	Pus culture: *B. pseudomellei*, hemoculture: NG, CT orbit: left panophthalmitis with subperiosteal abscess	I&D temporal space abscess, lateral and medial orbitotomy, I&D orbital abscess, IV ceftazidime then oral bactrim	VA 6/9, no sign of inflammation, residual ptosis, normal anterior segment, no record about posterior segment
				(onset = 3 weeks)											(improve)
2014	42	M	Farmer	Right eye contact with wood particle 10 days PTA then drop of breast milk into the eye 4 days PTA then acute visual loss 2 days, S/P IVT vancomycin, ceftazidime at provincial hospital	OD	HM at provincial hospital then no LP	Multiple keratic precipitates at the cornea, C/F 4+/4+, positive RAPD, vitreous opacity grade 4, B scan: loculated vitreous haze, membrane-like lesion attach to disc, moderate to high spike, intra-op finding: yellow pus with blood clot	Endogenous endophthalmitis	CKD, chronic alcoholism, wood particle contact, breast milk instillation	Multifocal	Eye	Splenic abscess	Gram stain from pus: gram-negative rod safety pin, pus culture: no growth, hemoculture: no growth, melioid titer 1:5122, ultrasound abdomen: splenic abscess	Enucleation, IV ceftazidime then oral bactrim	Good enucleation wound
				(onset = 10 days)											(enucleated)
2014	45	M	Officer	Proptosis 4 days PTA, S/P FESS, orbital decompression at private hospital	OS	CF	Marked eyelid swelling, fluctuation, chemosis, limit EOM at upper and lateral gaze, clear cornea, no C/F, negative RAPD, normal posterior segment, intra-op finding: loculated abscess at left upper eyelid 5 ml	Orbital cellulitis	DM without DR	Multifocal	Sinus	Abscess at right leg	Pus culture from the eye: *B. pseudomellei*, pus culture from the right leg: *B. pseudomellei*, hemoculture: no growth, ultrasound abdomen: no liver or splenic abscess	I&D, IV ceftazidime then oral bactrim	Less swelling, less chemosis, normal anterior segment, EOM improve, no record of VA and posterior segment
				(onset = 4 days)											(NA)
2015	50	M	Farmer	Low-grade fever 2 weeks PTA, right eye pain 9 days PTA, painful proptosis with visual loss 7 days PTA, S/P IV ceftazidime, IV metronidazole at provincial hospital, progressive proptosis in this admission	OD	HM	Proptosis, marked chemosis, AC deep with C/F 4+/3+, positive RAPD, peripheral synechiae, vitreous opacity grade 4, EOM minimal limit all direction, B scan: vitreous opacity, subretinal abscess, intra-op finding: yellow pus 0.2 ml	Panopthalmitis	–	Disseminated	Hematogenous	Liver abscess, ethmoid sinusitis	Hemoculture: no growth, melioid titer 1:5122, vitreous culture: no growth	PPV, IV ceftazidime	No LP, normal globe contour, no record about anterior and posterior segment
				(onset = 9 days)											(worse)
2015	45	M	Farmer	Painful proptosis with fever 2 weeks, S/P IV antibiotic at primary care hospital	OD	1/60 at primary care hospital then LP	Marked eyelid and periorbital area swelling, vesicle at medial canthus, marked bloody chemosis, clear cornea, no C/F, positive RAPD, EOM 0% all direction, necrotic skin at forehead 2 × 3 cm	Orbital cellulitis, Necrotizing fasciitis	DM without DR, psoriasis, chronic alcoholism	Disseminated	Eye	Sinusitis, septic arthritis, splenic abscess, septic shock	Hemoculture: *B. pseudomellei* x II, pus culture from the eye: *B. pseudomellei*, pus culture from the right knee: *B. pseudomellei*	I&D, FESS, skin debridement, IV ceftazidime	VA no LP, chemosis, normal anterior segment, RAPD positive, no record of posterior segment
				(onset = 2 weeks)											(worse)

Baseline characteristics of the patients were comparable to general melioidosis patients. The male to female ratio was 3 to 1 with a median age of 50.5 years old (39–70). The most common occupation was farmer (nine cases, 56%). Most patients had underlying diseases (14 cases, 88%), of which diabetes mellitus was the most common (12 cases, 75%). Ocular involvement was part of dissemination in nine cases (56%), which were classified as disseminated septicemic melioidosis.

The majority of ocular melioidosis patients (10 cases, 63%) presented with eye symptoms. Interestingly, the other six cases initially presented with fever or a headache. Out of the 15 cases for which there were records of visual acuity, 11 (73%) presented with blindness. The ocular manifestations of melioidosis were classified as orbital cellulitis (seven cases, 44%), preseptal cellulitis (two cases, 13%), endophthalmitis (four cases, 25%), panophthalmitis (two cases, 13%), and panuveitis (one case, 6%).

In most cases, the definitive management was surgery (13 cases, 81%) including incision and drainage, debridement (eight cases, 62%), pars plana vitrectomy (three cases, 23%), and enucleation (two, 15%). There were only three cases (19%) in which the patients were able to be treated without surgery.

Despite adequate surgical intervention, the visual outcomes of ocular melioidosis were disappointing. Out of the 14 cases for which there were records of final visual acuity, nine (64%) patients ended up legally blind. Three of these patients (20%) presented with no light perception at the beginning, two had to be enucleated, two (14%) were stable, and two (14%) had progressive loss of vision. Patients had improved vision after treatment in only five cases (36%).

## Discussion

To our knowledge, this is the first and largest case series of ocular involvement in melioidosis. A comprehensive literature review revealed only 14 cases from 12 reports [3–14], including 7 cases of orbital cellulitis (50%), 3 cases of endophthalmitis (21%), 3 cases of corneal ulcer (21%), and 1 case of acute dacryocystitis (7%). Most of the reports were single-case reports, and the largest one had only three cases.

In Thailand, especially in the northeast, there has been an increase in the reported cases of melioidosis. This is likely due to increasing awareness of the condition and increased sensitivity of the technology used to detect the organism. The mortality rate in these areas is around 40%. It is the third highest cause of mortality after acquired immune deficiency syndrome and tuberculosis [2]. Although ocular involvement in melioidosis is rare, the effects on patients’ vision are devastating. Most patients with this condition ended up becoming legally blind. In our series of 16 cases, there were only 5 (36%) in which patients had improved vision after treatment.

We suspect that the number of ocular melioidosis cases might be underestimated. Most of the melioidosis patients admitted to the hospital had disseminated septicemic melioidosis and were treated for life-threatening symptoms. Mild ocular symptoms might be easily overlooked, and ophthalmologists were not consulted in all cases.

The prevalence of ocular melioidosis in Srinagarind Hospital (1.02%) was about twice that in Khon Kaen Hospital (0.49%). The discrepancy might be due to the differences between the two hospitals. Srinagarind Hospital is the largest university hospital in northeast Thailand, and many severe cases of systemic melioidosis are referred to Srinagarind Hospital. Since more organs are affected in severe disseminated melioidosis, ocular involvement is more likely in these cases.

We suspect that the recent prevalence of ocular melioidosis in Srinagarind Hospital might be much higher than what we have found. From 2007 to April 2016, there were 264 cases of melioidosis at Srinagarind Hospital, of which 13 had ocular involvement. According to this finding, the prevalence during this time interval was as high as 4.9% (95% confidence interval from 2.82 to 8.32%).

This study led to some interesting findings. Ten patients (63%) presented with eye symptoms, which later resulted in systemic spreading. On the other hand, there were six patients (38%) whose first symptoms were not eye symptoms; four patients (25%) presented with fever and two (13%) presented with a headache. In most cases, diabetes mellitus was the underlying disease (12 cases, 75%), but none of the patients in those cases had diabetic retinopathy.

Interestingly, we found that most cases of ocular melioidosis were classified as disseminated septicemic melioidosis (nine cases, 56%) which means that there was a bloodstream infection. This is unlike other gram-positive organisms, which usually cause orbital cellulitis and commonly result in a negative hemoculture. The explanation for this finding may be attributable to the nature of *Bulkholderia pseudomallei* infection, which generally presents with bloodstream infection.

In our study, orbital cellulitis was the most common manifestation (seven cases, 44%). Usually, orbital cellulitis is caused by gram-positive organisms and can be cured only by intravenous antibiotics, unlike orbital cellulitis caused by melioidosis. All of these patients ended up undergoing surgical intervention (100%). The abscess-forming activity of *Burkholderia pseudomallei* may be the reason why intravenous antibiotics alone did not work to treat the condition.

Moreover, there were two cases (29%) of orbital cellulitis that progressed to necrotizing fasciitis, which is uncommon in other types of bacterial orbital cellulitis. This is similar to the results of a previous case report by Saonanon P [13]. Unfortunately, all of our patients (100%) with necrotizing fasciitis subsequently developed septic shock. Early suspicion and prompt surgical debridement may improve mortality in these patients.

We also found that even if systemic ceftazidime was used, the occurrence of endogenous endophthalmitis caused by melioidosis was not preventable, as stated in a previous report [10]. Most of the cases diagnosed as endophthalmitis and panophthalmitis required surgical intervention (five out of six cases, 83%), including pars plana vitrectomy (three out of five cases, 60%) and enucleation (two out of five cases, 40%).

Two cases (50%) of endophthalmitis were enucleated. The first case, from 2011, had a delayed presentation. The patient had experienced loss of vision for 1 month prior to admission, which was the longest onset in any of the cases. In the second case, from 2014, the patient exhibited two risk factors for the condition, including wood particle contact and breast milk instillation into the eye, as a result of local traditional treatment practices.

There were three cases that were cured without any surgical intervention. In one case, this was due to the patient seeking early treatment for endogenous endophthalmitis. The other two patients had diagnoses that did not require an operation (namely, panuveitis and preseptal cellulitis).

## Conclusions

In summary, ocular involvement in melioidosis was rare, but the outcomes were devastating. The most common ocular involvements were orbital cellulitis and endophthalmitis. The morbidity in these cases was high, so it is critical to employ a high index of suspicion. Ocular melioidosis should be considered when the ocular infection does not respond to conventional antibiotic therapy, especially in hyperendemic regions for melioidosis. Early consultation with an ophthalmologist and prompt surgical intervention may significantly improve the final visual outcomes, as well as mortality rates.

## Methods

We retrospectively reviewed all admitted cases of melioidosis with ocular involvement from two tertiary hospitals in Khon Kaen using electronic databases. The first is Srinagarind Hospital, which is a university hospital. We searched the hospital’s electronic database for cases of this condition from January 1993 to April 2016 (23 years and 4 months). The second is Khon Kaen Hospital, which is a provincial hospital. We searched the hospital’s electronic database for cases that presented between January 2012 and April 2016 (4 years and 4 months). The data were retrieved using the ICD10 code for melioidosis (all A24 codes) and all diseases of the eye and adnexa (code H00 to H59).

This manuscript adheres to the guidelines and principles laid out in the Declaration of Helsinki. Institutional review board (IRB) approval was obtained from the Khon Kaen University and Khon Kaen Hospital, Thailand. The clinical trial was registered in Thai Clinical Trials Registry (study ID: TCTR20160818004).

We only included cases in which there were positive cultures for melioidosis or high blood titer according to indirect hemagglutination (IHA). The cutoff point for positive antibody titers has been determined to be 1:160 in endemic areas [15]. Irrelevant ocular diagnoses, such as cataracts, glaucoma, diabetic retinopathy, or other underlying eye diseases, were excluded. The prevalence and 95% confidence intervals (95% CI) were calculated using the modified Wald method. Other results were summarized as proportions and percentages.

## Abbreviations

AC: Anterior chamber
*B. pseudomellei*: *Bulkholderia pseudomallei*
C/F: Cell/flare
CF: Counting fingers
CKD: Chronic kidney disease
DM: Diabetes mellitus
DR: Diabetic retinopathy
ECCE: Extracapsular cataract extraction
EOM: Extraocular movement
F: Female
FESS: Functional endoscopic sinus surgery
HM: Hand motion
I&D: Incision and drainage
IOP: Intraocular pressure
LP: Light perception
LP: Lumbar puncture
M: Male
MDS: Myelodysplastic syndrome
MRI: Magnetic resonance imaging
NA: Not available
OD: Right eye
OS: Left eye
PJ: Light projection
PPV: Pars plana vitrectomy
RAPD: Relative afferent pupillary defect
RD: Retinal detachment
RRAPD: Reverse relative afferent pupillary defect
VA: Visual acuity
